# Retraction Note: Flawless polyaniline coating for preservation and corrosion protection of ancient steel spearheads: an archaeological study from military museum, Al-Qala, Egypt

**DOI:** 10.1038/s41598-024-82504-6

**Published:** 2024-12-11

**Authors:** Mohamed M. Megahed, Noha H. Elashery, Saleh M. Saleh, Ashraf M. El-Shamy

**Affiliations:** 1 Conservation Department, Faculty of Archaeology, Fayoum University, Faiyum, Egypt; 2 Physical Chemistry Department, Electrochemistry, and Corrosion Laboratory, National Research Center, El-Bohouth St. 33, Dokki, P.O. 12622, Giza, Egypt

Retraction of: *Scientific Reports* 10.1038/s41598-024-57184-x, published online 27 March 2024

The Editors have retracted this Article.

After publication, concerns were raised regarding the data presented in three of the figures. Specifically:

- In Figure 2, Panel a appears to partially overlap with Panel b. These panels are described as originating from different samples.
- In Figure 3, Panel b reports a scale of 200µm and a Working Distance (WD) of 11.3mm, but the same image in Figure 5 indicates a scale of 500µm and a WD of 11.7mm.

In addition, concerns were raised regarding the accuracy of a number of the references used in the Article. The Editors therefore no longer have confidence in the reliability of the results and conclusions presented.

The Authors disagree with this retraction.

